# Small-vessel Disease in a Patient with Atrial Flutter: Unveiling a Potential Vascular Mechanism of Tachycardia-induced Cardiomyopathy

**DOI:** 10.19102/icrm.2026.17023

**Published:** 2026-02-15

**Authors:** Sebastian Weyand, Karin Klingel, Peter Seizer

**Affiliations:** 1 Medizinische Klinik II—Kardiologie und Angiologie, Ostalb-Klinikum Aalen, Aalen, Germany; 2 Allgemeine und Molekulare Pathologie und Pathologische Anatomie, Universitätsklinikum Tübingen, Tübingen, Germany

**Keywords:** Atrial flutter, heart failure, small-vessel disease, tachycardia-induced cardiomyopathy

## Abstract

A 65-year-old man presented with new-onset exertional dyspnea. The predominant abnormality was a marked concentric thickening of the arteriolar walls with strong smooth muscle actin positivity, consistent with small-vessel disease (SVD). This case illustrates a potential mechanism of tachycardia-induced cardiomyopathy (TIC), in which SVD may contribute to impaired myocardial perfusion and reversible left ventricular dysfunction under persistent tachycardia.

## Case presentation

**Figure 1:**
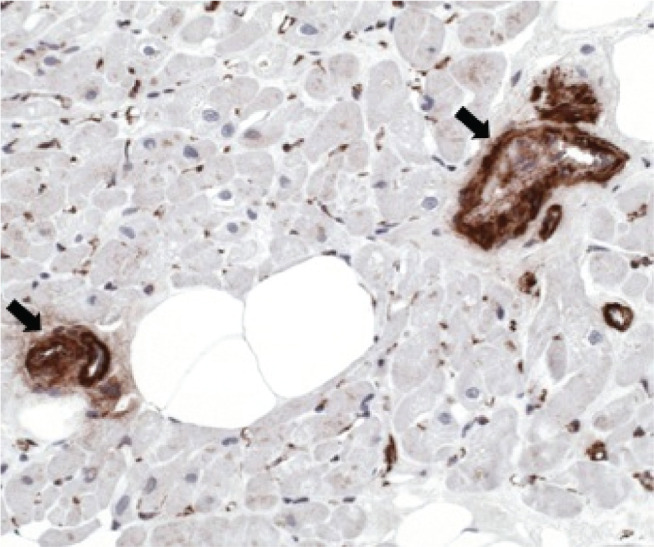
Endomyocardial biopsy showing arterioles with a marked concentric wall thickening (black arrows) and strong smooth muscle actin positivity in immunohistological stainings, consistent with small-vessel disease. The surrounding myocardium shows preserved architecture without inflammatory infiltrates or interstitial fibrosis, supporting the diagnosis of noninflammatory, microvascular remodeling.

A 65-year-old man presented with new-onset exertional dyspnea. No other symptoms were reported. An electrocardiogram showed a typical atrial flutter with 2:1 conduction. The patient reported having had a flu-like illness in the recent past. Echocardiography revealed a globally reduced left ventricular ejection fraction (LVEF) of 30% with normal wall thickness (interventricular septum, 11 mm; posterior wall, 10 mm) and no signs of left ventricular outflow tract obstruction. After ruling out coronary artery disease, an endomyocardial biopsy was performed as part of the diagnostic workup for newly diagnosed nonischemic cardiomyopathy with severely reduced left ventricular function of unclear etiology. Myocarditis was excluded because histological and immunohistological examination showed neither inflammatory infiltrates nor myocyte injury. Hypertrophic cardiomyopathy was ruled out based on preserved myocardial architecture, without myocyte disarray or interstitial fibrosis. Instead, the predominant abnormality was a marked concentric thickening of the arteriolar walls with strong smooth muscle actin positivity **(Figure 1)**, consistent with small-vessel disease (SVD). Eight weeks after successful atrial flutter ablation, the patient was asymptomatic and had an improved LVEF of 50%.

Informed consent was obtained from the patient prior to the procedure.

## Discussion

The present case illustrates a potential mechanism of tachycardia-induced cardiomyopathy (TIC), in which SVD may contribute to impaired myocardial perfusion and reversible left ventricular dysfunction under persistent tachycardia.

Histopathological studies of TIC, as summarized by Mueller et al.,^1^ have demonstrated that this condition is characterized by distinct structural and cellular alterations. Endomyocardial biopsies typically reveal mild interstitial fibrosis; hypertrophied cardiomyocytes with preserved overall architecture; and a macrophage-dominated, low-grade inflammatory pattern with enhanced expression of major histocompatibility complex class II molecules but only minimal T-cell infiltration. Electron microscopy and immunohistochemistry further show mitochondrial remodeling with redistribution of mitochondria toward the intercalated disks (the “EMID” sign) and evidence of metabolic adaptation, including increased expression of the mitochondrial pyruvate carrier 1, supporting heightened energetic demand under sustained tachycardia. Experimental models of TIC have also demonstrated activation of neurohormonal pathways such as the renin–angiotensin–aldosterone system, along with matrix remodeling and altered calcium handling, which contribute to reversible myocardial dysfunction.

Unlike the low-grade, macrophage-predominant inflammatory pattern described in the histopathological studies of TIC, our patient’s biopsy did not show relevant inflammatory infiltrates by CD3+ T-cells and CD68+ macrophages. Cardiomyocyte architecture was preserved, myocyte necrosis was absent, and interstitial fibrosis was minimal. Instead, the predominant finding was a marked arteriolar wall thickening with strong smooth muscle actin positivity, consistent with SVD. This pattern suggests that, in the absence of inflammatory remodeling, microvascular dysfunction may have increased the vulnerability to tachycardia-induced systolic dysfunction.

The detection of SVD in this case emphasizes the importance of considering microvascular dysfunction as a possible underlying mechanism in the diagnostic workup of patients with TIC, especially when other causes of heart failure have been ruled out. SVD may reduce myocardial microvascular reserve and thereby increase susceptibility to tachycardia-related systolic dysfunction. Although this finding does not currently alter clinical management beyond rhythm control, recognizing SVD as a contributing factor may help explain interindividual differences in the reversibility of myocardial dysfunction and could, in the future, open perspectives for adjunctive therapies targeting microvascular or endothelial dysfunction.

While the exact mechanisms linking SVD and TIC are not fully understood, this case supports the hypothesis that microvascular dysfunction can facilitate reversible ventricular impairment under sustained tachycardia. Experimental studies have shown that chronic supraventricular tachycardia reduces myocardial blood flow despite preserved epicardial coronaries,^2^ a vulnerability that may be accentuated in the presence of SVD. The recovery of systolic function after rhythm control in our patient, despite histological evidence of SVD, supports a predominantly functional rather than structural mechanism of myocardial dysfunction. Although causal inference cannot be drawn from a single observation, these findings provide pathophysiological plausibility for SVD as a potential co-factor in TIC. Further studies are warranted to explore the contribution of microvascular dysfunction to disease expression and recovery in this context.
